# An evolutionary perspective on night terrors

**DOI:** 10.1093/emph/eoy010

**Published:** 2018-04-14

**Authors:** Sean D Boyden, Martha Pott, Philip T Starks

**Affiliations:** 1 Department of Biology, Tufts University, Medford, MA 02155, USA; 2 Eliot-Pearson Department of Child Study and Human Development, Tufts University, Medford, MA 02155, USA

**Keywords:** parasomnia, physiological benefits, psychosocial benefits, diseases of culture, sleep training

## Abstract

Night terrors, also known as sleep terrors, are an early childhood parasomnia characterized by screams or cries, behavioral manifestations of extreme fear, difficulty waking and inconsolability upon awakening. The mechanism causing night terrors is unknown, and a consistently successful treatment has yet to be documented. Here, we argue that cultural practices have moved us away from an ultimate solution: cosleeping. Cosleeping is the norm for closely related primates and for humans in non-Western cultures. In recent years, however, cosleeping has been discouraged by the Western medical community. From an evolutionary perspective, cosleeping provides health and safety benefits for developing children. We discuss night terrors, and immediate and long-term health features, with respect to cosleeping, room-sharing and solitary sleeping. We suggest that cosleeping with children (≥1-year-old) may prevent night terrors and that, under certain circumstances, cosleeping with infants (≤11-months-old) is preferable to room-sharing, and both are preferable to solitary sleeping.

## INTRODUCTION

Night terrors are an early childhood parasomnia associated with disturbance from non-REM, slow-wave sleep [1]. According to the American Academy of Sleep Medicine’s (AASM) *International Classification of Sleep Disorders*, night terrors (also known as sleep terrors) are defined as ‘a cry or piercing scream, accompanied by autonomic nervous system and behavioral manifestations of intense fear. … Sometimes there is prolonged inconsolability associated with a [night] terror’ [2]. Notably, night terrors are distinguishable from less severe nightmares by difficulty in waking the child [3]. These events are stressful and disturbing for the child experiencing them, the parents of the child and other family members [4].

The prevalence of night terrors in children is difficult to assess. Research has yielded discrepant results regarding the likelihood of experiencing night terrors with measurements ranging from 1.7% to almost 56% of individuals and ages ranging from 18 months to adolescence [4–6]. (Night terrors also occur in adults, but rarely so.) Prevalence has most frequently been assessed in school-age children, although research has demonstrated that night terrors are most common in children between 1 and 5 years of age [1, 4, 5]. The difficulties in determining prevalence are probably due to the varying definitions of night terrors used in research studies. This underscores a broader finding by Hublin and colleagues that the general public does not have one clear definition of night terrors, and thus often confuses them with simple nightmares [7]. Because of this, we use the AASM’s definition of night terrors provided above [2].

Attempts to treat night terrors have yet to establish an effective remedy for the condition. Currently, the recommended treatment for night terrors is to leave the child alone; parents are encouraged to let the terror proceed uninterrupted, as the child is unlikely to respond to attempts to be woken and is often inconsolable upon awakening [4]. Some sedative medications have proven effective in case studies [8, 9]; however, such medications may lead to tolerance or dependence in children, and the notion of treating children with sedatives is troubling to many. Scheduled awakenings, performed around the time when the child transitions from non-REM to REM sleep, have also been used as treatment for night terrors [10].

One practice that has not been investigated in the context of night terrors is cosleeping. One of us (PTS) has a personal anecdote on this topic. By the summer of 2014, PTS’ 3-year-old child had been experiencing four to seven night terrors weekly for several months. Finding this behavior disturbing, PTS standardized the child’s schedule, modified his diet, and monitored for suitability the images and stories to which he was exposed. When these failed to have any discernable effect, PTS subjected the child to scheduled awakenings, which similarly had little impact. Finally, and possibly due to exhaustion, PTS began cosleeping with the child; the child’s night terrors rapidly ceased and have not reoccurred.

Cosleeping is not a novel behavior. Cosleeping is observed in all closely related primates and in many current human societies. It is traditionally defined as caregivers sharing a bed with offspring, and this is the definition we use here. In addition to the potential benefit to *children* (≥1-year-old) suggested by the anecdote above, cosleeping has demonstrated physiological effects on *infants* (≤11-months-old) [11, 12]. Moreover, the impact on a mothers’ sleep duration or stage of sleep is negligible [13]. In spite of this, cosleeping with infants has been advised against by the American Academy of Pediatrics [14].

Here, we present an argument that night terrors are the result of an environmental mismatch between evolved behavior and the modern cultural practice of solitary sleeping. Using an environmental mismatch approach, attachment theory and research on the physiological, behavioral and psychosocial impacts of cosleeping, we argue that cosleeping is beneficial for children and may prevent or greatly reduce night terrors. We further suggest that under certain circumstances cosleeping with infants is preferable to room-sharing, and both are preferable to solitary sleeping.

## HISTORICAL VERSUS MODERN ENVIRONMENTS: THE MISMATCH HYPOTHESIS

In recent years, the environmental mismatch hypothesis—the idea that specific traits evolved to maximize their fitness in an environment very different from the one in which they are expressed today—has become a growing model for the study of the evolutionary basis of disease [15]. Many disorders have been studied through an environmental mismatch lens. One popular example is obesity, a particularly concerning condition due to its links with diabetes and cardiovascular disease. Several environmental mismatch hypotheses have been proposed for the rising prevalence of obesity; for example, genes linked with increased fat storage would have been evolutionarily favorable for Paleolithic hunter-gatherers, for whom food was not always readily available. However, as humans progressed into developed, sedentary societies with consistent access to food, this adaptation has become a pathology: average body weight and obesity have increased throughout the world [16].

The mismatch approach has also been applied to the study of human sleep behavior and sleep disorders. Significant changes to human sleep patterns have occurred throughout evolutionary time, including a decrease in the amount of time humans sleep relative to other primates [17]. Such changes have been explored as potential factors in sleep disorders ranging from insomnia to sleep apnea [18]. Additional research has shown an increase in sleep disorders in recent years; this increase has been partially attributed to the increase in light pollution and constant mental stimulation of developed societies [15, 19, 20].

The mismatch approach also provides a rational and plausible explanation for night terrors. The benefits of a child sleeping with a parent were likely manifold in the evolutionary environment in which humans evolved—most obvious is the decreased risk of predation of children if parents were nearby. Indeed, when school-aged children have any memory of a night terror they typically report indistinct recollections of threats (such as monsters, spiders, snakes, etc.) or fear of ‘something’ that ‘is after me’ or ‘that is going to get me’ [21]. Other benefits, such as shared body heat and greater physiological regulation (e.g. of the heartbeat) have been demonstrated in infants (see Table 1 [11, 22–27]). Thus, night terrors may be an extreme response to the evolutionary-environmental mismatch that has resulted from changes in human sleeping behavior from cosleeping to sleeping separately.

**Table 1.** The likely relative behavioral and physiological effects of solitary sleeping, cosleeping and room-sharing between a caregiver and an infant (≤11-months-old)^a^

Effect	Solitary sleeping	Cosleeping	Room-sharing	References
Behavioral				
Caregiver responsivity	–	↑	↑	[22, 23]
Breastfeeding rate	–	↑	↑/–	[24]
Smothering risk	–	↓/–	–	[25]
Physiological				
Thermoregulatory development	–	↑	–	[26]
Respiratory regulation	–	↑	–	[27]
Ease of arousal	–	↑	↑/–	[26]
Unexpected infant death risk	–	↑	↑/–	[11, 27]

a (Proper bedding and surrounds are assumed for all conditions.) Solitary sleeping is considered the standard (–). ↑ indicates a beneficial effect, ↓ indicates a detrimental effect and – indicates no change in situation for the infant relative to the standard. Multiple designations are provided when the outcome is in question. From a cost-benefit approach, solitary sleeping fairs very poorly and cosleeping is preferable whenever the risk of smothering (purple) is lower than the added benefits over room-sharing (orange).

## ATTACHMENT THEORY

The practice of cosleeping is also supported by attachment theory, which addresses the prolonged period of helplessness in human infants and the infant’s need to elicit the mother’s (or other caregiver’s) protection and care [28]. These behaviors are rooted in evolution, providing a survival advantage by increasing caregiver-infant proximity. They include infant rooting and signaling (e.g. crying) and caregiver responsivity (meeting the infant’s need) and sensitivity (meeting the need in a timely fashion). The attachment system is activated in the presence of stress, either internally or externally derived. Evidence of the system can be seen in the first few weeks of life, when the infant begins signaling and the caregiver responds. It peaks at about 1 year of age, the time when the child typically develops independent locomotion and can get away from the mother, and continues at high intensity throughout the years of dependency in early childhood [29]. It is of interest that the age range during which attachment behaviors are strongest is the age range when night terrors first present [4, 5]. Indeed, one Swiss study found that cosleeping, while uncommon in children below 1 year of age (<10%), increased during ages when night terrors are most common [30].

### Primates and cosleeping: an ancient and modern practice

Cosleeping is observed in all closely related primates, as well as a significant portion of human populations. Barry and colleagues collected data on sleeping arrangements for 90 cultures, and found that mother and infant slept in the same bed in 41 of them (46%); mother slept in the same room with the infant but in an unspecified bed in 30 (33%), and in the same room in separate beds in 19 (21%). In none of 90 cultures did the mother and infant sleep in a separate room [31]. Despite this, cosleeping has been discouraged by the American Academy of Pediatrics due to a stated link with sleep-related infant deaths [25]. Research on the rates of cosleeping in the US has shown that, although cosleeping increased from 6.5% to 13.0% from 1993 to 2010, no significant increase was observed in white families from 2001 to 2010; these findings suggest that recommendations against cosleeping are not uniformly followed across cultural groups [32].

Cosleeping has, however, persisted in small-scale, high-fertility/high-mortality cultures that characterized human societies for much of our evolutionary history. A study on the Aka hunter-gatherers and Ngandu farmers of central Africa by Hewlett and Roulett found that an overwhelming majority of offspring coslept with their parents from infancy through adolescence, although rates decreased as children aged [33]. Reasons for cosleeping include limitations in space, protection from predators and shared heat sources (e.g. body heat), similar adaptive benefits that likely promoted cosleeping in early human evolution [33].

Attitudes about cosleeping are beginning to change on a broad scale in Western nations. In particular, the UK, which formerly held views similar to those in the US that discourage cosleeping, has begun to embrace parents’ choice to cosleep [34]. Overall, the UK has become more open to parents’ decisions on infant cosleeping; the same cannot be said for the US [14, 35]. Data suggest that this may be unfortunate.

The adaptive benefits of cosleeping in humans can easily be seen through the physiological effects on parent and infants [27]. We summarize some of these and other findings on the study of different sleeping practices on parent and infant behavior and physiology in Table 1. Although the American Academy of Pediatrics has reported risks of cosleeping over solitary sleeping or room-sharing, including overheating and smothering [25], they may have overemphasized these risks or ignored factors contributing to them [36]. In addition, the reported risks may be balanced by the benefits provided by cosleeping, including improvements in thermoregulation and respiratory regulation, increased breastfeeding and easier arousal of both infants and parents (see Table 1).

In addition to the benefits in infancy, there are numerous developmental and psychosocial benefits for individuals who coslept as children that persist into adulthood. As reviewed by McKenna and Gettler [37] and summarized here in Table 2 [38–41], studies of children who coslept found that they were less dependent on their parents compared to their solitary sleeping peers. In studies of college-aged populations, individuals who reported cosleeping with their parents in childhood (between the ages of 2 and 13) had higher self-esteem, increased comfort with sexuality, and greater overall life satisfaction. It appears that cosleeping has no lasting negative repercussions [42] and may have significant long-lasting benefits.

**Table 2.** The relative psychosocial effects of solitary sleeping and cosleeping between a caregiver and child (ages ranging from 2 to 13 years of age across studies)^a^

Effect	Solitary sleeping	Cosleeping	Reference
Independence from parents	–	↑	[38]
Self-esteem in adulthood	–	↑	[39, 40]
Comfort with sexuality in adulthood	–	↑	[39]
Life satisfaction in adulthood	–	↑	[41]

a Solitary sleeping is considered the standard (–). ↑ indicates a beneficial effect and – indicates no change in situation for the child relative to the standard. We have excluded room sharing due to the lack of data and to difficulties predicting outcomes. Cosleeping appears to be preferable to solitary sleeping.

For many families, solitary sleeping has replaced cosleeping, and is often accomplished through the practice of sleep training. Sleep training involves parents leaving an infant or child alone in a separate room at night, and limiting responsiveness to its cries to the point of extinction, thus encouraging it to self-settle [43]. These practices are arguably disturbing for the infant or child, who is responding to the separation from its caregiver, and the cries are stressful for parents [44]. Although very popular and widely recommended [45], sleep training is counterintuitive to attachment theory and other evolutionary tenets of a responsive parent-offspring relationship.

Although formally a proponent of solitary sleeping [14], the American Academy of Pediatrics has recently revised its recommendations for safe infant sleep to include room-sharing, but not bed-sharing, of the parents and infant [25]. These recommendations posit that room-sharing with an infant increases the ability of the parent to quickly respond to the infant, while minimizing the risk of suffocation or overheating. These revisions are a move in the right direction; however, cosleeping in a safe environment appears to have increased benefits on infant (Table 1), and thus could offer more benefits than simply sharing the same room. The difference in short-term (Table 1) and long-term (Table 2) benefits between cosleeping, room-sharing, and solitary sleeping merits increased attention from sleep researchers.

We propose that cosleeping with children may reduce or possibly prevent night terrors. Accordingly, we are referring to the post-infancy stage with respect to this parasomnia. We include the infancy data, however, because there appear to be benefits for cosleeping with infants and because cosleeping with infants is likely to lead to cosleeping with children. It is important to note that not all children develop night terrors, and many are able to sleep in rooms separate from their parents nightly. The argument presented in this commentary is not, however, to suggest that night terrors will *always* result from separation of a parent and child at night; rather, we propose night terrors to be an extreme outcome of this separation, one that likely works together with other physiological and/or psychosocial factors.

## EXTENSIONS, TESTABLE PREDICTIONS AND LIMITATIONS

Several testable predictions, both observational and interventional, arise from the argument made here. One observational prediction is that children who cosleep will have a lower prevalence of night terrors compared with children who sleep solitarily. A comparison between the prevalence of night terrors in children who cosleep and children who sleep in the same room with parents should be undertaken to determine the degree of physical proximity with the parent necessary to influence positive change, should it occur. Previous research suggests that cosleeping and room-sharing have beneficial effects for infants (see Table 1), but it is unclear if either approach is superior with respect to parasomnias in young children.

One interventional prediction of our hypothesis is that children with night terrors would experience a decrease in incidence once they began cosleeping with their parents. There are currently no findings on the correlation between these phenomena beyond our anecdotal report (see above), but an interventional, prospective study could easily test this prediction. However, possible complications could arise as a result of the lack of understanding surrounding night terrors in the lay public and scientific community alike [7]. Any study undertaken to assess cosleeping as an intervention for night terrors would have to take care in defining night terror symptoms (intensity, duration, etc.) as well as defining cosleeping (bed-sharing vs room-sharing). We are currently beginning work on such a study.

### Alternative hypotheses

We have presented a mechanistic hypothesis with an evolutionary basis: the lack of cosleeping could reasonably trigger night terrors. This does not necessarily mean that cosleeping currently provides a fitness advantage.

One could hypothesize that solitary sleeping fosters a child’s independence from its parents. Fitness benefits may accrue from this if children who learn separation from their parents earlier display better adjustment or self-reliance in adulthood. We do not favor this hypothesis because, to our knowledge, there is no evidence that solitary sleeping leads to better adjustment. In fact, attachment theory suggests the opposite; caregiver sensitivity and responsivity lead to secure attachment in children. Secure attachment is associated with child compliance (increases safety), increased and better social relationships (survival and reproductive advantage) [28, 29]. Long-term psychosocial benefits due to cosleeping, which are indicative of secure attachment, can be found in Table 2.

One could hypothesize a direct fitness cost to cosleeping: cosleeping may present significant risk to the child in the form of accidental smothering while the parent is sleeping. This is the core argument the medical field uses against cosleeping [25]. We do not favor this hypothesis for caregiver-child cosleeping: we could not locate any studies of accidental smothering deaths in children beyond infancy.

Cosleeping may present a direct fitness cost for infants. It is unclear, however, how great this risk is or how costly the absence of cosleeping is to infants. Smothering rates are very low (0.1 per 1000 live births, as described in a New Zealand population study [46]), and many cases of smothering involve drug and/or alcohol abuse, or some other extenuating circumstance, that prevents the parent from waking up to their offspring’s cries or movements [47, 48, 49]. It may be that, for responsible, sober parents, the benefits to an infant for cosleeping with a caregiver on an appropriately designed bed outweighs the risk of smothering (Table 1). This warrants future exploration.

In short, we are unaware of any fitness benefit to the child (or parent) for solitary sleeping. Since our most closely related primates all cosleep with their young, and since individuals from many non-Western cultures cosleep with their young, solitary sleeping is clearly a culturally derived trait. At best, solitary sleeping may be selectively neutral, but data on SIDS rates and the American Academy of Pediatrics’ recent recommendations against it [25], suggest that solitary sleeping is maladaptive.

## CONCLUSION

To our knowledge, this paper is the first to propose that night terrors are an extreme response to a novel environment by children who sleep apart from their parents. We have addressed the many benefits of cosleeping to the infant (Table 1), and this paper extends the argument into the early childhood years (Table 2), showing that physiological and psychosocial benefits for infants (i.e. survival, protection) give way to physiological and psychosocial benefits for young children (i.e. survival, protection and relational dependence). This bio-behavioral scaffolding is precisely what contributes to the child feeling protected and thus safe in a sleeping environment that includes the caregiver. Cosleeping, however, is currently discouraged in Western cultures. Further research is needed to understand if the discouragement is warranted. It may be that a return to cosleeping practices in Western cultures will lead to better child health overall, including a reduction in the prevalence of night terrors.
